# Control of multi-joint arm movements for the manipulation of touch in keystroke by expert pianists

**DOI:** 10.1186/1471-2202-11-82

**Published:** 2010-07-14

**Authors:** Shinichi Furuya, Eckart Altenmüller, Haruhiro Katayose, Hiroshi Kinoshita

**Affiliations:** 1 School of Science and Technology, Kwansei Gakuin University, Sanda, Japan; 2 Institute of Music Physiology and Musicians Medicine, Hanover University of Music and Drama, Hanover, Germany; 3 School of Science and Technology, Kwansei Gakuin University, Sanda, Japan; 4 Graduate School of Medicine, Osaka University, Toyonaka, Japan

## Abstract

**Background:**

Production of a variety of finger-key touches in the piano is essential for expressive musical performance. However, it remains unknown how expert pianists control multi-joint finger and arm movements for manipulating the touch. The present study investigated differences in kinematics and kinetics of the upper-limb movements while expert pianists were depressing a key with two different touches: pressed and struck. The former starts key-depression with the finger-tip contacting the key, whereas the latter involves preparatory arm-lift before striking the key. To determine the effect of individual muscular torque (MUS) as well as non-muscular torques on joint acceleration, we performed a series of inverse and forward dynamics computations.

**Results:**

The pressed touch showed smaller elbow extension velocity, and larger shoulder and finger flexion velocities during key-depression compared with the struck touch. The former touch also showed smaller elbow extension acceleration directly attributed to the shoulder MUS. In contrast, the shoulder flexion acceleration induced by elbow and wrist MUS was greater for the pressed touch than the struck touch. Towards the goal of producing the target finger-key contact dynamics, the pressed and struck touches effectively took advantage of the distal-to-proximal and proximal-to-distal inter-segmental dynamics, respectively. Furthermore, a psychoacoustic experiment confirmed that a tone elicited by the pressed touch was perceived softer than that by the struck touch.

**Conclusion:**

The present findings suggest that manipulation of tone timbre depends on control of inter-segmental dynamics in piano keystroke.

## Background

A key issue in motor control research has been to understand how the nervous system organizes redundant number of joints for a purposeful movement production [1]. To elucidate this, a number of studies have investigated skillful multi-joint arm movements, such as reaching toward the target [2,3], drawing a line or circle [4], throwing the ball [5,6], and striking the piano key from some height [7-9]. A common observation was that while moving the arm toward the target, the motor system took advantage of the inter-segmental dynamics originating from proximal muscular contraction to accelerate distal joint rotation effectively [2,5,9]. However, these studies have focused on gross movement where proximal muscles are mainly used for movement production [10,11]. Little has been known about the organization of multi-joint movements during fine motor actions that are performed predominantly by distal muscles.

A comparative study of piano keystrokes with pressed and struck touches may be of help to better understand this issue. The former touch starts with the finger-tip contacting with the key, followed by an instantaneous production of force to depress the key. The latter touch involves a preparatory lifting of the arm, followed by a downswing before the finger-tip collides and depresses the key. In a series of biomechanical studies, we have recently examined kinematics, kinetics, and muscular activities of the upper-limb movements during the struck touch performed by expert pianists [7-9,12]. Results demonstrated that during downswing the pianists volitionally decelerated shoulder extension to generate the inter-segmental dynamics that accelerates elbow and wrist joint rotations. They also produced shoulder flexion motion during key depression, and thereby increased the angle of the finger segment relative to the key ("attack angle"). This resulted in the decreased finger joint torque that was produced by the key-reaction force, which lessened finger muscular force for compensating it. In contrast to these, information on the pressed touch has been fairly limited. Some researchers have examined kinematics and kinetics of the key movements while pianists were striking a key using the pressed and struck touches at various sound dynamics [13,14]. Results showed that the profiles of key reaction force in these touches clearly differed across all levels of tone loudness. The pressed touch was characterized by a less steep initial force development with smaller fluctuations in the subsequent period than the struck touch, implying precise control of the finger-tip movements. To our knowledge, however, no information on differences in the organization of the upper-limb movement during keystroke by the pressed and struck touches has been characterized. Understanding this is of significance not only for illustrating multi-joint movement control during fine motor actions such as tool-use, but also for some practical reasons. For example, previous psychoacoustic studies have shown these two touches elicited tones with different perception of timbre [15,16], suggesting a possibility of determining the movement organization responsible for manipulating tone timbre through examining these two touches. There is also clinical importance since a number of studies have reported playing-related injuries among piano players worldwide, but its risk factors remain unclear [17,18].

The primary purpose of the present study was to determine differences in kinematics and kinetics of the upper-limb movements during key depression between the pressed and struck touches. We specifically focused on the effect of preparatory arm-downswing on the cause-and-effect relation between motor commands issued into muscles and kinematics of key-depressing motion. To this aim, we used an induced acceleration analysis, which consists of inverse and forward dynamics computations [19,20]. This technique allows for quantitatively determining the effect of individual muscular torque (MUS) as well as non-muscular torques on accelerating all the joints in the motor system. We hypothesized that no preparatory arm downswing in the pressed touch would result in larger elbow extension and wrist flexion accelerations directly attributed to their corresponding MUS, and larger shoulder and finger flexion velocities as well as their accelerations induced by the corresponding MUSs, as compared to the struck mode. The second purpose of the study was to determine differences between perception of timbre of the tone generated by the pressed touch and the struck touch by using a psychoacoustic experiment. Previous studies showed that the timbre of tones produced by these two touches could be differentiated by ordinal subjects [15,16], but it remains unknown what kind of perception would be evoked by listening to these tones.

## Methods

### Participants

Seven right-handed expert pianists (3 males and 4 females, mean age ± SD = 24.3 ± 3.2 years) with more than 15 years of classical-piano training participated in the biomechanical experiment, and another eight pianists with more than 10 years of piano playing (4 males and 4 females, mean age ± SD = 25.8 ± 4.9 years) participated in the perceptual test. All the expert pianists who participated in the biomechanical experiment had won awards at domestic and/or international classical piano competitions. In accordance with the Declaration of Helsinki, the experimental procedure was explained to all participants and each participant signed a written informed consent. The study was approved by the local ethics committee at Osaka University.

### Experimental apparatus and key-striking task

The experimental apparatuses used were a Yamaha U1 upright acoustic piano, two 2-D position sensor systems (C5949, Hamamatsu Photonics Co., Japan), a sound-level meter (NA-27, Rion Co., Japan), and a stereo sound amplifier. In the C3-key, a strain-gauge miniature uniaxial force transducer was installed at its distal end. All signals from the position sensor, the amplifier of the force transducer, and the sound-level meter were stored on a PC via a 12-bit A/D converter with a sampling frequency of 900 Hz.

The experimental task was a right-hand keystroke of the C3 key by the middle finger in a short tone production mode (*staccato*) with struck and pressed touches. For the struck touch, each participant began with the fingertip lightly touching the key, lifted his/her right arm/hand to a self-determined height, and struck the key at a designated level of tone. For the pressed touch, each participant depressed the key with the finger initially resting on the key surface. In both touches, the participant then lifted his/her hand and arm again as a follow-through, and returned to the initial position. The left arm was kept relaxed and placed on the side of the trunk while the trunk was in an upright position with minimal movement.

Two loudness levels of *piano *(p) and *forte *(f), which corresponded to maximum key-force levels of 4.4 and 9.6 N, respectively, were selected in this study. Kinematic, kinetic and simultaneous sound data were collected from 30 successful strokes at each target loudness level with a strike-to-strike interval of approximately 10 s for each participant. The target loudness level was a prerecorded piano sound on a minidisk, which was presented from a set of speakers placed on top of the piano. The subject was asked for a retrial when the sound pressure level (SPL) of the elicited tone was greater or smaller by more than 0.9 dB compared with the target SPL.

### Data acquisition procedure

The movement of the upper-limb in the sagittal plane was recorded using one of the position sensor cameras (sampling freq. = 150 Hz). The LEDs required for this purpose were mounted on the skin over the fingertip of the middle finger and at the centers of the metacarpo-phalangeal (finger), styloid process (wrist), head of radius (elbow), and coracoid process (shoulder) joints. The data was digitally smoothed at a cut-off frequency of 12 Hz using a second-order Butterworth digital filter. The angular displacement at the finger, wrist, elbow, and shoulder joints, and that of the middle finger segment relative to the key surface were then numerically computed.

The C3-key kinematics was recorded using another position sensor camera and an LED placed on the key surface. The onset of the key descending movement ("the finger-key contact moment") was determined when the calculated vertical velocity of the key exceeded 5% of its peak.

### Induced acceleration analysis

We used an induced acceleration technique that consists of a sequence of inverse and forward dynamics computations [19,20]. An advantage of this technique is that it allows us to quantitatively determine the effect of torque produced at one joint on movements at the other joints in the multi-segmental motor system (i.e. "inter-segmental dynamics"). Using the measured kinematic and key-force data along with the anthropometric data of each participant, inverse dynamics computation were initially performed to obtain the time varying muscular torques (MUS) at the shoulder, elbow, wrist, and finger joints. The MUS value was calculated as follows:

$$MUS=I(\theta )\ddot{\theta }-V(\theta ,\dot{\theta })-J^{T}(\theta )F$$

Note that we defined MUS as muscular torque that removed static component counteracting with gravitational torque, which is directly used for limb movement production [9,21]. For the purpose of this study, the upper-extremity was assumed as four interconnected rigid links (upper arm, forearm, hand, and finger) (see details in [9]). Complete equations of motion are listed in the Additional file 1.

Based on the computed MUS values, we then used forward dynamics equations to compute angular accelerations induced by the MUS as follows:

$$\ddot{\theta }=I{\left(\theta \right)}^{-1}(MUS+V(\theta ,\dot{\theta })+J^{T}(\theta )F)$$

This equation tells us that the angular acceleration at each joint is produced by MUS at shoulder, elbow, wrist and finger joints, velocity-dependent torque (VEL), and reaction-force torque originating from mechanical interaction between the finger-tip and key (REA).

To evaluate kinetic source of the angular velocities produced during key depression (between the moment of finger-key contact and the moment of the key's lowest position), we then computed the integral of the shoulder, elbow, wrist and finger joint accelerations produced by each MUS ($imp{\ddot{\theta }}_{MUS}$), VEL ($imp{\ddot{\theta }}_{VEL}$), and REA ($imp{\ddot{\theta }}_{REA}$) from the moment of finger-key contact (T1) to the moment of lowest key position (T2) as follows.

$$\begin{array}{l} imp{\ddot{\theta }}_{MUS}=\int_{T1}^{T2}I{\left(\theta \right)}^{-1}MUS\;dt\\ imp{\ddot{\theta }}_{VEL}=\int_{T1}^{T2}I{\left(\theta \right)}^{-1}v\text{(}\theta \text{,}\dot{\theta }\text{) }dt\\ imp{\ddot{\theta }}_{REA}=\int_{T1}^{T2}I{\left(\theta \right)}^{-1}J^{T}\text{(}\theta \text{)}F\;dt \end{array}$$

### Perceptual test of tone timbre

A discrimination task of tone's timbre difference was separately performed. To obtain the sound for this test, one expert pianist was asked to strike the C3 key of a grand piano (Bösendorfer 225) in a sound-proof room with the struck and pressed touches. The generated sound was recorded using a dynamic microphone (BETA58a, SHURE co.) and an audio interface (EDIROL UA-4FX, Roland co.) with the sampling frequency of 44.1 kHz. In total, twenty sounds were recorded for each touch, and from these, four tones (2 with struck, 2 with pressed) with the same loudness level (110dB: *mf*) and same duration was carefully selected. Another eight pianists were then asked to listen to these tones, and rate (1) the quality of tone (rich/plane) and (2) attack (hard/soft) of each tone using visual analog scale (-3 (most plane/hard) to 3 (most rich/soft)). They could listen to each stimulus as often as wanted until they were sure about their judgments.

### Statistical data analysis

Using touch and loudness as independent variables, a two-way ANOVA with repeated measures was performed for each of the dependent variables (p < 0.05).

## Results

### Touch-dependent difference in endpoint and joint kinematics

**Figure 1:**
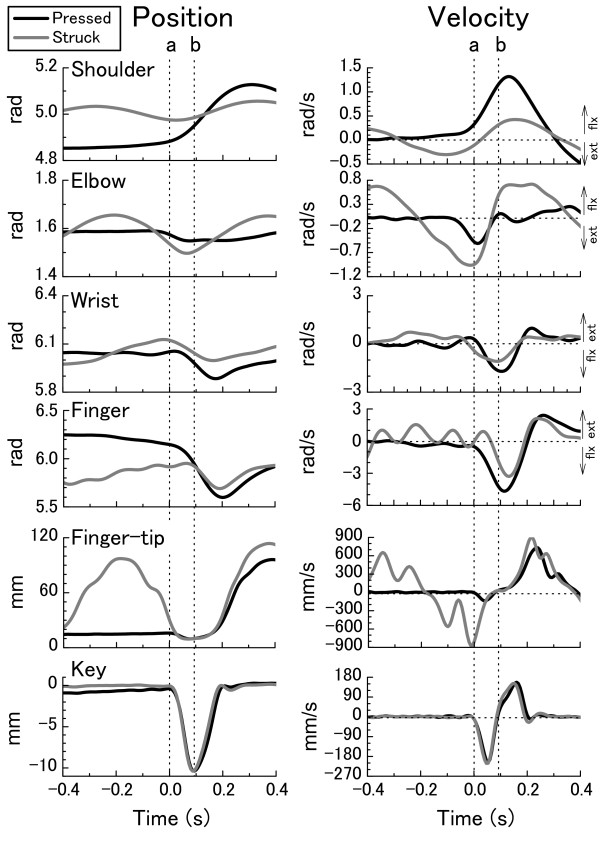
**The time-history curves of the shoulder, elbow, wrist, and finger joint angles, finger-tip and key's vertical positions (left panel), and their velocities (right panel) at the *forte *loudness level with pressed (black line) and struck touches (gray line) by one representative pianist**. The curves represent the average of 30 keystrokes. The dotted vertical lines indicate the moment of finger-key contact (**a**), and the moment of the lowest key position, when the key-depression was ended (**b**).

Figure 1 illustrates mean time-history curves of joint angles, finger-tip and key's vertical displacement (left panel), and their derivatives (right panel) at *forte *across 30 trials with the pressed and struck touches in one representative participant. When initiating key-depression, the shoulder joint was less extended, and the finger joint was less flexed during the pressed touch as compared to the struck touch. During the key-depressing phase, flexion at the shoulder, wrist and finger joints, and extension at the elbow joint occurred in both touches. However, during the pressed touch, flexion at the shoulder and finger joints displaced much larger than the struck touch, resulting in similar joint angles in both touches at the end of key-depression.

**Figure 2:**
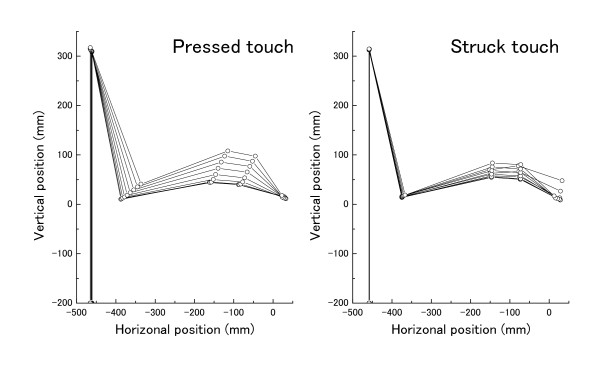
**Stick figures of the upper-limb when one representative pianist was striking at the *forte *loudness level with the pressed (left panel) and struck (right panel) touches during the finger-key contacting period (from the moment of finger-key contact to the moment of finger-key release)**.

These features are also illustrated by the stick picture of the upper-limb during the finger-key contacting period (from the moment of finger-key contact to the moment of finger-key release) in Figure 2. While depressing the key, the upper- and fore-arms moved forward and upward, and the hand moved forward and downward in both touches. These forward and rotational movements of the upper-limb were much greater during the pressed touch than the struck touch.

Table 1 lists the group means of kinematic variables for all participants at each loudness level. ANOVA revealed that the peak finger-tip velocity was significantly smaller at louder tone and during the pressed touch than the struck touch. The peak key velocity was significantly greater at *forte *than *piano*. The angles of the attack and the finger joint at the key-contact moment were significantly smaller at the pressed touch compared with the struck touch. As for the values at the lowest key position, none of the joint angles showed significant effects of touch, loudness, and their interaction.

**Table 1:** Mean values of finger-tip and key velocities, and joint angles at the finger-key-contact and key-bottom moments

	Pressed		Struck		ANOVA results		
	
Variables	*piano*	*forte*	*piano*	*forte*	Touch **F**^**(1,6)**^	Loudness **F**^**(1,6)**^	Touch × Loudness **F**^**(1,6)**^
**Peak descending velocity (mm/s)**							
Finger-tip	466(292)	576(299)	578(275)	748(238)	6.6 *	14.1 **	0.5
Key	268(119)	330(142)	213(34)	317(91)	5.2	80.1 **	7.0

**Joint angle at key contact (rad)**							
Attack angle	1.80(0.16)	1.80(0.22)	1.92(0.13)	1.91(0.18)	9.6 *	0.0	0.3
Finger	6.16(0.09)	6.15(0.08)	6.02(0.13)	6.01(0.13)	18.9 **	0.3	0.2
Wrist	6.15(0.03)	6.16(0.03)	6.17(0.08)	6.18(0.05)	1.4	1.5	0.1
Elbow	1.43(0.13)	1.43(0.13)	1.45(0.16)	1.47(0.15)	4.9	1.9	6.0*
Shoulder	4.91(0.04)	4.89(0.10)	4.92(0.10)	4.93(0.10)	3.5	0.0	3.4

**Joint angle at key lowest (rad)**							
Attack angle	1.90(0.21)	1.88(0.24)	1.87(0.32)	1.89(0.38)	0.1	0.0	2.6
Finger	6.08(0.13)	6.09(0.11)	6.09(0.06)	6.07(0.09)	0.0	0.3	1.4
Wrist	6.14(0.10)	6.12(0.10)	6.17(0.09	6.17(0.08)	4.8	2.0	1.6
Elbow	1.41(0.10)	1.41(0.11)	1.40(0.11)	1.39(0.10)	4.4	1.0	0.3
Shoulder	4.95(0.08)	4.94(0.08)	4.92(0.10)	4.94(0.10)	1.1	0.2	1.0

The numbers in the parenthesis indicate the standard deviation. * p < 0.05, ** p < 0.01

**Figure 3:**
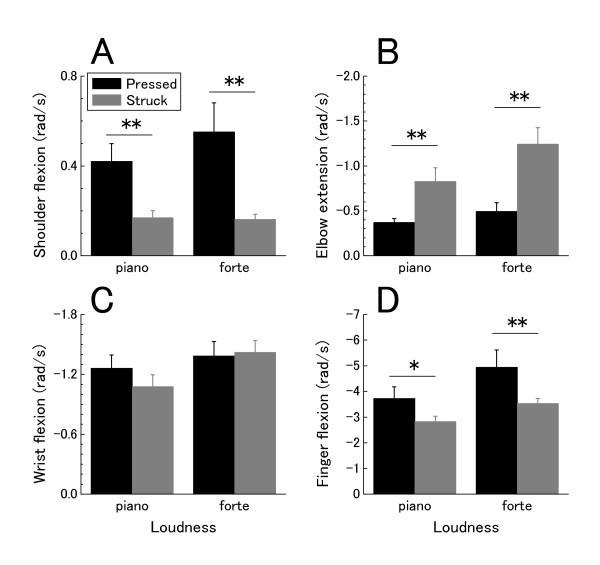
**The group means of the peak angular velocities for shoulder flexion (A), elbow extension (B), wrist flexion (C), finger flexion (D) at two loudness levels**. Error bars represent ± 1 SE.

Figure 3 shows the means for the peak angular velocities of shoulder flexion, elbow extension, wrist flexion, and finger flexion for all participants during key depression. It is clear from the means that the peak elbow velocity was smaller during the pressed touch compared with the struck touch. The peak shoulder and finger velocities were, on the other hand, greater during the pressed touch. ANOVA revealed a significant main effect of touch for the velocities of shoulder (F(1, 6) = 13.2, p < 0.05), elbow (F(1, 6) = 15.6, p < 0.01), and finger (F(1, 6) = 6.4, p < 0.05). There was also a significant loudness × touch interaction effect for the elbow velocity (F(1, 6) = 14.6, p < 0.01). Elbow extension velocity was smaller during the pressed touch than the struck touch, but the difference was much greater at louder tone. Neither loudness × touch interaction nor touch effects were found for the wrist joint.

### Induced acceleration analysis

**Figure 4:**
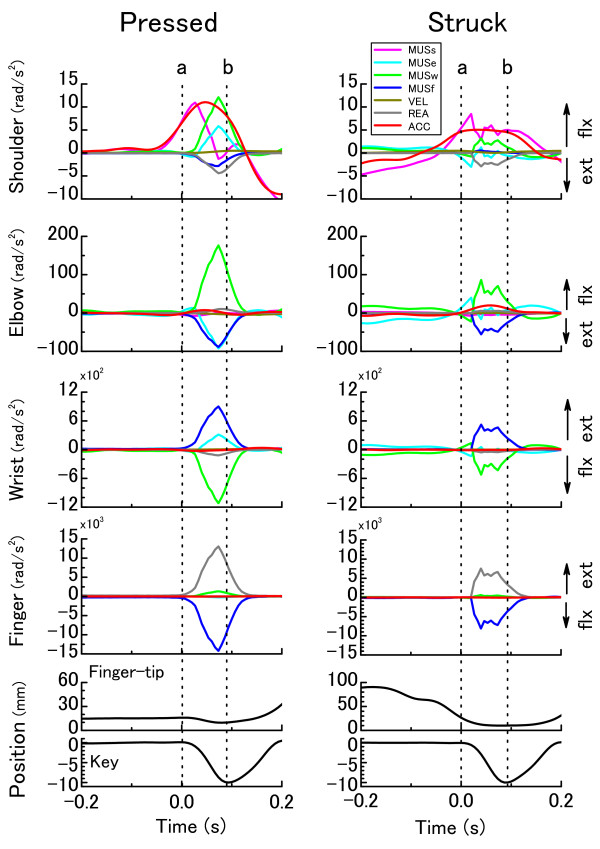
**The time-history curves of net joint acceleration (ACC in red) and its six components at the shoulder, elbow, wrist, and finger joints, and key and hand vertical position at the *forte *loudness level when one representative pianist were striking the key with the pressed (left panel) and struck (right panel) touches**. MUSs, MUSe, MUSw, MUSf, VEL, and REA corresponded to joint acceleration attributed to shoulder MUS (violet), elbow MUS (right blue), wrist MUS (right green), finger MUS (navy blue), VEL (ocher), and REA (grey), respectively. The curves represent the average of 30 keystrokes. The dotted vertical lines indicate the moments of finger-key contact (**a**) and lowest key position (**b**).

Figure 4 shows representative mean time-history curves of angular accelerations at the shoulder, elbow, wrist and finger joints (ACC), those attributed to shoulder, elbow, wrist, and finger muscular torques (MUSs, MUSe, MUSw, MUSf), velocity-dependent torque (VEL), and key-reaction torque (REA), and vertical position of the finger-tip and key during the pressed (left panel) and struck (right panel) touches.

There was marked development of shoulder flexion acceleration during key depression for both modes of key touch. For the pressed touch, a clear increase in flexion acceleration attributed to shoulder MUS was initially observed, which followed by increases in elbow and wrist MUS. For the struck touch, there was an increase in flexion acceleration attributed to shoulder MUS. While both of the two touches showed extension acceleration attributed to REA, acceleration by VEL was indiscernible.

For both modes of key touch, elbow joint accelerations induced by wrist and finger MUS exhibited a counteractive relationship during the finger-key contacting period. The pressed touch also showed marked extension acceleration induced by elbow MUS during the period of key-depression.

For both modes of key touch, flexion acceleration at the wrist joint during key depression was induced by wrist MUS, which was commonly counteracted by finger MUS. However, the magnitudes of these were greater during the pressed touch than the struck touch. Only for the pressed touch, there were small but substantial amount of extension acceleration attributed to elbow MUS during the period of key-depression.

During the key-depression period, finger joint acceleration induced by finger MUS was always counteracted in both timing and magnitude by the REA. The magnitudes of these were apparently greater at the pressed touch compared with the struck touch.

### Identification of joint toques contributing to joint acceleration

A limitation of Fig. 4 was that the large scale of y-axis made it difficult to visually evaluate which joint torques contributed to the total joint acceleration. Since our aim of the kinetic analysis was to clarify dynamics underlying kinematics differences between the two touches (i.e. Fig.3), we attempted to quantitatively determine joint torques contributing to acceleration for shoulder flexion, elbow extension, wrist flexion and finger flexion by means of inverse dynamics analysis. We first decomposed each of six computed joint torques into two subcomponents, either of which was the same (*T *^+^) or opposite (*T *^-^) sign with the net torque proportional to joint acceleration responsible for key-depression. We then computed a contribution index (CI) by dividing the impulse of one subcomponent having the same sign with the net torque during the key-depression period by the sum of impulses of the two subcomponents. That is,

Accordingly, the CI represented how much an individual torque contributed to the acceleration. If the value was close to 1, the torque mostly contributed to the joint acceleration during key depression. The equations of motion used in the inverse dynamics were shown previously [9].

Table 2 lists the group means of CI values for MUSs, MUSe, MUSw, MUSf, VEL, and REA at shoulder, elbow, wrist, and finger joints at each loudness level. For the shoulder, MUSs, MUSe, MUSw, and VEL showed relatively high CI values for the pressed touch, whereas only MUSs and VEL showed apparently high values for the struck touch. ANOVA revealed that the pressed touch had smaller value for MUSs and MUSf and greater value for the distal MUSe and MUSw compared with the struck touch. For the elbow, MUSe, MUSf, and VEL commonly exhibited high CI values in both the pressed and struck touches. In addition, the CI value of MUSs for the struck touch was significantly higher than that for the pressed touch, confirming utilization of the proximal-to-distal inter-segmental dynamics. For the wrist, MUSs, MUSw, and REA showed high CI values in both two touches. Also, MUSe was an additional contributor to the acceleration only for the struck touch. For the finger, the MUSs, MUSe, MUSf, and VEL showed relatively greater CI values in both two touches.

**Table 2:** Means of contribution index (CI) for individual torque at shoulder, elbow, wrist and finger joints

	Pressed				Struck				ANOVA results		
	
Variables	*piano*		*forte*		*piano*		*forte*		Touch **F**^**(1,6)**^	Loudness **F**^**(1,6)**^	Touch × Loudness **F**^**(1,6)**^
**Shoulder**											
MUSs	**0.60**	(0.41)	0.47	(0.35)	**0.79**	(0.34)	**0.80**	(0.27)	13.27 *	1.38	4.77
MUSe	**0.81**	(0.27)	**0.88**	(0.24)	0.43	(0.28)	0.45	(0.30)	12.47 *	2.02	0.57
MUSw	**0.74**	(0.33)	**0.71**	(0.37)	0.18	(0.26)	0.22	(0.33)	9.50 *	0.12	2.91
MUSf	0.09	(0.15)	0.11	(0.17)	0.24	(0.37)	0.41	(0.37)	6.05 *	2.14	1.08
VEL	**0.81**	(0.33)	**0.81**	(0.33)	**1.00**	(0.00)	**1.00**	(0.00)	2.40	N/A	N/A
REA	0.00	(0.00)	0.00	(0.00)	0.00	(0.00)	0.00	(0.00)	0.07	4.13	0.03

**Elbow**											
MUSs	0.46	(0.35)	0.47	(0.28)	**0.70**	(0.32)	**0.65**	(0.30)	7.62 *	0.49	0.02
MUSe	**0.80**	(0.32)	**0.79**	(0.32)	**0.71**	(0.30)	**0.61**	(0.28)	0.74	1.77	1.82
MUSw	0.03	(0.06)	0.04	(0.09)	0.07	(0.13)	0.02	(0.03)	0.13	0.38	0.74
MUSf	**0.79**	(0.31)	**0.81**	(0.24)	**0.68**	(0.30)	**0.70**	(0.29)	1.02	0.13	0.00
VEL	**0.90**	(0.25)	**0.90**	(0.25)	**1.00**	(0.00)	**1.00**	(0.00)	1.00	N/A	N/A
REA	0.14	(0.36)	0.03	(0.05)	0.12	(0.19)	0.13	(0.28)	0.88	2.78	0.24

**Wrist**											
MUSs	**0.57**	(0.28)	**0.54**	(0.26)	**0.56**	(0.46)	**0.67**	(0.44)	0.11	0.35	1.25
MUSe	0.06	(0.08)	0.04	(0.07)	**0.54**	(0.29)	**0.62**	(0.22)	57.60 *	0.22	1.17
MUSw	**0.88**	(0.25)	**0.89**	(0.25)	**0.90**	(0.16)	**0.90**	(0.10)	0.02	0.15	0.04
MUSf	0.00	(0.00)	0.00	(0.00)	0.00	(0.00)	0.02	(0.06)	1.37	1.04	1.04
VEL	0.00	(0.00)	0.00	(0.00)	0.00	(0.00)	0.00	(0.00)	N/A	N/A	N/A
REA	**0.81**	(0.33)	**0.81**	(0.32)	**1.00**	(0.00)	**1.00**	(0.00)	2.43	0.38	1.87

**Finger**											
MUSs	**0.52**	(0.35)	0.43	(0.16)	**0.58**	(0.41)	**0.66**	(0.30)	1.98	0.01	1.04
MUSe	**0.70**	(0.32)	**0.80**	(0.32)	0.44	(0.27)	0.44	(0.28)	4.05	1.19	2.06
MUSw	0.03	(0.07)	0.02	(0.05)	0.02	(0.02)	0.01	(0.01)	0.25	1.76	0.13
MUSf	**1.00**	(0.00)	**1.00**	(0.00)	**1.00**	(0.00)	**1.00**	(0.00)	0.36	0.39	1.93
VEL	**0.68**	(0.33)	**0.68**	(0.33)	**0.61**	(0.36)	**0.58**	(0.34)	0.10	0.07	0.26
REA	0.00	(0.00)	0.00	(0.00)	0.00	(0.00)	0.00	(0.00)	1.01	0.82	0.99

Bolded numbers indicate the value greater than 0.5.

The numbers in the parenthesis indicate the standard deviation. * p < 0.05

N/A: CI value for all subjects showed 1 or 0, and thus there was no between-subject variance

### Impulse of joint acceleration produced by its surrounding muscles

**Figure 5:**
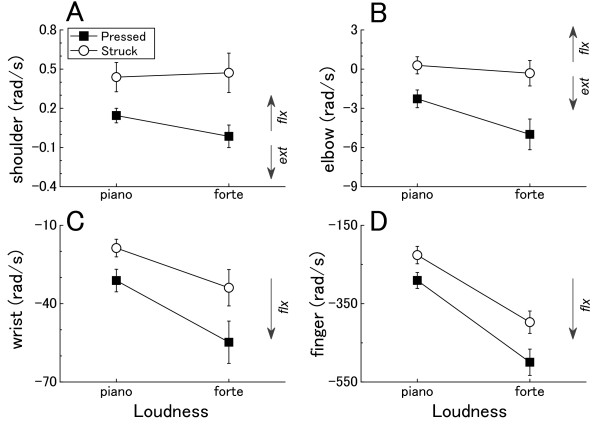
**The group means of the **$imp{\ddot{\theta }}_{MUS}$** for the shoulder (A), elbow (B), wrist (C) and finger (D) joints at each loudness level**. * p < 0.05, ** p < 0.01. The error bars represent ± 1 SE. A positive value indicates flexion for the shoulder and elbow joints, and extension for the wrist and finger joints (see arrows).

Figure 5 A-D shows the impulse of angular acceleration attributed to each MUS ($imp{\ddot{\theta }}_{MUS}$) at the shoulder, elbow, wrist and finger joints at each loudness level. ANOVA revealed that the pressed touch had a significantly smaller $imp{\ddot{\theta }}_{MUS}$ for the shoulder flexion (F(1, 6) = 12.1, p < 0.05), and larger $imp{\ddot{\theta }}_{MUS}$ for the elbow extension (F(1, 6) = 41.1, p < 0.01), wrist flexion (F(1, 6) = 178.6, p < 0.01), and finger flexion (F(1, 6) = 93.3, p < 0.01) compared with the struck touch. The group × loudness interaction was also significant in this variable at the shoulder (F(1, 6) = 10.6, p < 0.05), elbow (F(1, 6) = 23.0 p < 0.01), and wrist joints (F(1, 6) = 10.8, p < 0.05). The interaction effect indicated that with the generation of louder sound, $imp{\ddot{\theta }}_{MUS}$ at the shoulder joint decreased, and $imp{\ddot{\theta }}_{MUS}$ increased at the elbow and wrist joints for the pressed touch. The loudness effect was significant at the elbow, wrist and finger joints.

### Impulse of joint acceleration by muscular torques at the adjacent joints

Table 3 lists the group means of impulse values for the shoulder, elbow, wrist and finger joint accelerations directly attributed to MUS generated at their adjacent joints at each loudness level. In the following impulse analyses (Tables 3 and 4), we only present results of the torques mostly contributing to joint acceleration during key depression, which were determined in the former CI analysis (see Table 2).

**Table 3:** Means of impulse of shoulder, elbow, wrist and finger joint accelerations attributed to MUS at the adjacent joints

	Pressed		Struck		ANOVA results		
	
Variables	*piano*	*forte*	*piano*	*forte*	Touch **F**^**(1,6)**^	Loudness **F**^**(1,6)**^	Touch × Loudness **F**^**(1,6)**^
**Imp. of shoulder joint accel. (rad/s)**							
elbow MUS	0.279(0.221)	0.604(0.376)	0.067(0.196)	0.176(0.331)	31.4 **	12.7 *	19.8 **
wrist MUS	0.164(0.200)	0.324(0.304)	0.057(0.128)	0.106(0.224)	8.1 *	7.1 *	7.1 *
finger MUS	-0.031(0.066)	-0.073(0.115)	0.004(0.065)	0.022(0.078)	4.7	0.8	7.9 *

**Imp. of elbow joint accel. (rad/s)**							
shoulder MUS	-0.173(0.115)	-0.015(0.156)	-0.497(0.326)	-0.579(0.403)	9.7 *	1.6	11.7 *
finger MUS	-2.75(0.84)	-4.857(1.8)	-2.12(0.89)	-3.78(1.61)	25.8 **	34.3 **	1.7

**Imp. of wrist joint accel. (rad/s)**							
shoulder MUS	-0.087(0.177)	-0.043(0.089)	-0.163(0.442)	-0.193(0.446)	0.9	0.8	5.7
elbow MUS	8.03(5.15)	15.81(8.89)	1.64(4.80)	3.95(8.63)	44.8 **	10.5 *	23.9 **

**Imp. of finger joint accel. (rad/s)**							
shoulder MUS	-0.085(0.223)	-0.140(0.398)	-0.016(0.414)	-0.073(0.401)	0.2	1.6	0.0
elbow MUS	-5.87(2.35)	-11.61(4.81)	-0.17(4.53)	-1.92(6.34)	23.9 **	13.2 **	16.3 **

The numbers in the parenthesis indicate the standard deviation. * p < 0.05, ** p < 0.01

**Table 4:** Means of impulse of shoulder, elbow, wrist and finger joint accelerations attributed to VEL and REA

	Pressed		Struck		ANOVA results		
	
Variables	*piano*	*forte*	*piano*	*forte*	Touch **F**^**(1,6)**^	Loudness **F**^**(1,6)**^	Touch × Loudness **F**^**(1,6)**^
**Imp. of shoulder joint accel. (rad/s)**							
VEL	0.013 (0.011)	0.018 (0.015)	0.030 (0.025)	0.056 (0.037)	6.7 *	11.1 *	14.5 **

**Imp. of elbow joint accel. (rad/s)**							
VEL	-0.042 (0.057)	-0.057 (0.071)	-0.033 (0.031)	-0.061 (0.052)	0.0	8.2 *	8.7 *

**Imp. of wrist joint accel. (rad/s)**							
REA	-2.594 (0.858)	-4.431 (1.371)	-1.911 (0.492)	-3.139 (0.912)	20.9 **	51.7 **	6.2 *

**Imp. of finger joint accel. (rad/s)**							
VEL	-0.016 (0.025)	-0.049 (0.041)	-0.012 (0.030)	-0.015 (0.041)	6.0	2.5	4.5

The numbers in the parenthesis indicate the standard deviation. * p < 0.05, ** p < 0.01

At the shoulder joint, the pressed touch had significantly greater flexion velocity induced by elbow and wrist MUS compared with the struck touch. For the elbow, the pressed touch had significantly smaller and greater extension velocity by the shoulder MUS and finger MUS compared with the struck case, respectively. For all of these variables except for elbow extension acceleration by finger MUS, there was a significant interaction effect between the touch and loudness. The interaction effect indicates that with the generation of louder sound, the pressed touch had a greater increase in shoulder flexion acceleration by elbow and wrist MUS, smaller increase in elbow extension acceleration induced by shoulder MUS, and greater increase in this variable by finger MUS as compared to the struck touch. For the wrist and finger, the pressed touch showed greater extension and flexion acceleration induced by elbow MUS compared with the struck touch, respectively. These variables also showed an interaction effect, indicating a greater increase for the former touch with the generation of louder tone.

### Impulse of joint acceleration by velocity-dependent and reaction-force dependent torques

Table 4 lists the group means of impulse values for the shoulder, elbow, wrist and finger joint acceleration attributed to VEL and REA at each loudness level. For the shoulder joint, the pressed touch showed significantly smaller flexion velocity attributed to VEL compared with the struck touch, although its magnitude was fairly small. For the elbow joint, there was no significant difference in VEL between the two touches. For the wrist, the pressed touch showed greater flexion velocity by REA compared with the struck touch. A touch × loudness interaction effect was also found for the velocity produced by shoulder and elbow VEL, and wrist REA. The interaction effect indicated that with the generation of louder sound, the pressed touch showed a smaller increase in shoulder and elbow velocity by VEL, and greater increase in wrist velocity by REA as compared to the struck touch. For the finger, flexion acceleration by VEL did not differ between the two touches.

### Comparison of the impulse of joint acceleration across torques

**Figure 6:**
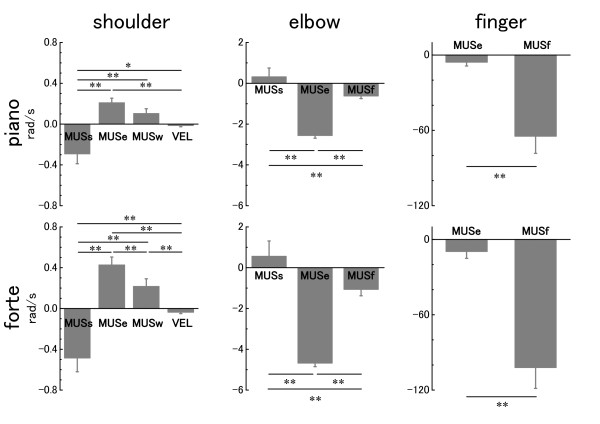
**A comparison of difference values of the impulse of joint acceleration attributed to individual torque between the pressed and struck touches across different torques for the shoulder, elbow and finger joints at each loudness level**. The negative value indicates that the impulse at the pressed touch is smaller for the shoulder, and smaller for the elbow and finger compared with the struck case. Note that a result of the wrist joint was not shown due to no significant difference in the peak angular velocity between the two touches. * p < 0.05, ** p < 0.01. The error bars represent ± 1 SE.

To assess the primary joint torques that are responsible for differences in joint velocity observed between the touches, we subtracted the impulse value of joint acceleration attributed to each torque for the struck touch from that for the pressed touch, and compared it across different joint torques (Figure 6). We performed this analysis only for the joints and torques with a significant difference between the touches in the impulse analyses (i.e. Fig.5, Tables 3 and 4). For the shoulder, the difference value between the touches was positive for elbow and wrist MUS (pressed > struck), and negative for shoulder MUS and VEL (pressed<struck). A torque × loudness two-way ANOVA with repeated measures revealed both effects of interaction (F(3, 18) = 17.33; p < 0.01) and torque (F(3, 18) = 17.76; p < 0.01). For the elbow, the value was positive for shoulder MUS (pressed < struck), and negative for elbow and finger MUS (pressed > struck). A repeated measures ANOVA found effects of loudness × torque interaction (F(2, 16) = 24.21; p < 0.01), torque (F(2, 16) = 40.03; p < 0.01), and loudness (F(1, 6) = 9.41; p < 0.05). Note that a relatively small difference value regarding shoulder MUS was because the present impulse analysis did not include the period before the finger-key contact [9]. For the finger, both the values for the finger and elbow MUS were negative (pressed>struck), and their difference was significant (ANOVA: F(1, 6) = 72.83; p < 0.01).

### Perception of the tone timbre

Results of the psychoacoustic experiment showed that tones generated by the pressed touch were perceived softer compared with those by the struck touch. The mean attack of tone for all participants was 1.0 ± 1.5 (SD) and -1.3 ± 1.0 for the pressed and struck touch, respectively. ANOVA revealed a significant touch effect on attack of tone (F(1, 15) = 28.3, p < 0.01). Concerning the quality of tone, their mean value was -0.4 ± 1.3 and 0.4 ± 1.5 for the pressed and struck touch, respectively. ANOVA revealed no significant touch-dependent difference on this variable.

## Discussion

In the present study, the pressed touch showed greater shoulder and finger flexion velocity, and smaller elbow extension velocity compared with the struck touch. For the shoulder, the pressed touch involved smaller flexion acceleration by shoulder MUS, and greater flexion acceleration by elbow and wrist MUS compared with the struck touch. The larger shoulder flexion velocity at the former touch therefore resulted mostly from greater effect of distal-to-proximal inter-segmental dynamics. For the elbow, the pressed touch had greater extension acceleration by elbow and finger MUS, and smaller extension acceleration by shoulder MUS than the struck touch. For the finger, the pressed touch showed substantially greater flexion acceleration by finger MUS compared with the struck touch, confirming that the former touch belongs to fine movements. Note that the acceleration produced by velocity-dependent torque was small relative to that produced by the other torques at all joints, which differs from large effects of velocity-dependent torque during the ball-throw [22]. This can be due to different movement speed between ball-throw (more than 25m/s) and piano keystroke (at most 1m/s). A psychoacoustic test further showed that the timbre of a tone elicited by the pressed touch was perceived softer than that by the struck touch.

### Kinematics and kinetic differences between the pressed and struck touches

Our kinematic analysis found smaller peak elbow extension velocity, and larger peak shoulder and finger flexion velocity when the pressed touch was used compared with when the struck touch was used. We previously found a drastic increase in elbow extension acceleration toward the end of downswing in the struck touch [7], which may explain smaller elbow extension velocity during key depression at the present pressed touch. In addition, our findings indicated that the pianists compensated for this insufficient elbow speed during the pressed touch by increasing shoulder and finger velocity in order to reach the finger-tip velocity for eliciting the target loudness of tone. We also found that the finger joint was extended more and attack angle was smaller at the onset of key depression for the pressed touch than the struck touch. This hand posture would provide the pressed touch with a longer horizontal distance from the joint center to the finger-tip compared with the struck touch, which consequently enhances effectiveness of finger flexion rotation for generating the descending motion of the finger-tip at the former touch. The present pianists may therefore also compensate for the lack of preparatory arm-downswing by taking advantage of the geometric configuration of the hand and finger for depressing the key during the pressed touch.

To understand the dynamics underlying these kinematic differences between the pressed and struck touches, we performed inverse and forward dynamics computations. The results indicated that greater shoulder flexion velocity during the pressed touch was mostly accounted for by greater shoulder flexion velocity induced by elbow and wrist muscular contractions. Effective use of the distal-to-proximal inter-segmental dynamics has been reported during skilled throwing and striking movements in the 3 D space [6,22,23]. For example, a skilled thrower exploited the distal-to-proximal inter-segmental dynamics from elbow muscular contraction for facilitating shoulder internal rotation, and thereby instantaneously accelerated forward motion of the hand just before releasing the ball [22]. However, to our best knowledge, there has been no study that addressed the use of the distal-to-proximal dynamics in the 2 D limb movements. This would be because the present pressed touch requires instantaneous acceleration at the finger-tip for generating the target key velocity due to strong spatio-temporal constraints, which is absent in tasks that have been investigated in previous studies (see reviews by [10,11]). The effective utilization of distal-to-proximal inter-segmental dynamics can thus be a specialized movement strategy the nervous system uses for instantaneously accelerating limb endpoint in skilled multi-joint movements.

A role of vigorous shoulder flexion during key depression has been also considered to increase the attack angle and configure upstanding finger posture for lessening finger joint torque produced by the key-force as well as muscular work compensating for it [8,24]. In the pressed touch, however, configuring such an upstanding finger posture seems to require different motor skill from the struck touch. This is firstly because compared with the struck touch the pressed touch initiated a key-depression with a relatively extended finger posture and smaller attack angle, and secondly because the pressed touch had smaller shoulder flexion acceleration generated by its surrounding muscles, which might be due to stabilizing work of shoulder muscles against the key-reaction force. Nevertheless, we found that both attack and finger joint angles at the end of key depression did not differ between the two modes of key touch. Therefore, during the pressed touch the effective use of distal-to-proximal inter-segmental dynamics for accelerating shoulder must also have played an essential role in configuring the mechanically-advantageous finger posture.

We also found that during key depression, the pressed touch had greater elbow extension acceleration attributed to elbow MUS than the struck touch. This is contradictory with the kinematic finding since the former touch showed smaller elbow extension velocity. A further analysis revealed that the pressed touch had smaller elbow extension acceleration by shoulder MUS than the struck one. These indicated that smaller elbow extension velocity during the former touch resulted mostly from the failure to fully exploit the effect of the proximal-to-distal inter-segmental dynamics. Similar to the elbow, production of wrist and finger flexion acceleration also relied more on their surrounding muscles during the pressed touch than the struck touch. It is logical to assume that the distal muscles of less strength and endurance are commonly more sensitive to fatigue than the proximal muscles, and therefore stronger reliance on distal muscles with the pressed touch suggests that it is less efficient than the struck touch. A significant interaction effect of loudness and touch on many kinematic and kinetic variables examined further suggests that differential effects of the distal muscular work between the two touch modes become greater at the production of a louder tone

Playing the piano involves a repetition of keystroke reaching sometimes thousands of times per minute [25]. Repetitive submaximal muscular efforts may create cumulative damage in muscles and tendons, especially in the hand and forearm over times. Indeed, researchers have reported that more than 60% of active piano players at some time experience playing-related injuries from acute pain to more serious symptoms such as tendonitis and focal dystonia [17,18]. The present finding of greater reliance on the distal muscles in the pressed touch than the struck touch particularly for stronger keystroke may therefore emphasize importance of avoiding repetitive use of the former touch for eliciting a loud tone in order to prevent the injury.

### Manipulation of the timbre

To be able to elicit tones with variations in the timbre is an indispensable motor skill in musical performance. A recent psychoacoustic study showed that listening to musical stimuli having the same acoustic characteristic except for the timbre evoked different emotional experience to listeners [26], emphasizing a significance of manipulating tone timbre for expressive musical performance. Relating to the piano, studies have shown that the timbre of tones produced by the struck and pressed touches could be differentiated by ordinal subjects [15,16]. Our study extended these by showing that a piano tone produced by the pressed touch was perceived softer than that by the struck touch. This timbre difference would be related to various factors, including touch-dependent differences in the noise due to the finger-key collision [13,14] and in key-force profile [14], and nonuniform dynamic property of the finger-tip pulp [27]. In addition, previous findings of audio-visual interaction in the perception of tone [28] suggest that kinematic differences between the present two touches may also evoke different tone perception to audience in a live performance. Hence, in spite of relative inefficiency over the struck touch, the pressed touch would be an indispensable motor skill for pianists to accomplish expressive musical expression. The present biomechanical and psychoacoustic findings would further provide novice piano players and piano teachers with implications that soft tone timbre could be produced by initiating key-depression with relatively extended finger posture and accentuating shoulder-finger flexion rotations.

### Task-relevant control of the inter-segmental dynamics

Dounskaia [10] recently proposed a leading joint hypothesis that provided comprehensive explanation for the control of multi-joint movements. In this framework, there is one (leading) joint that generates the inter-segmental dynamics at the adjacent (subordinate) joints. The subordinate joints utilize and/or compensate for the inter-segmental dynamics while accounting for its timing and magnitude so as to fulfill task requirements. Evidence in favor of this idea has been provided in various skilled multi-joint movements [2,4-6,9]. The present findings also supported it since the pianists effectively utilized the inter-segmental dynamics for accelerating the limb during both pressed and struck touches. The contrasting relation of leading and subordinate joints between the two touches further implies that production of a variety of tone timbre in piano playing depends on control of the inter-segmental dynamics. Since accurate control of the inter-segmental dynamics requires its precise internal representation [3], manipulation of the tone timbre may need to develop distinct and multiple internal representations of the limb dynamics, such as the MOZAIC model [29,30].

## Conclusions

The present study probed into control strategy of multi-joint finger and arm movements for dexterous manipulation of the way of depressing a key by expert pianists. We specifically examined two different but fundamental touches in piano playing; pressed and struck. The most striking finding was that the pianists performed a key-depression primarily by utilizing the distal-to-proximal and proximal-to-distal inter-segmental dynamics during the pressed and struck touches, respectively. A psychoacoustic test further revealed differences in perception of the timbre of a tone elicited by these two touches. Taken together, these findings indicate that manipulation of tone timbre in piano playing depends on control of the inter-segmental dynamics.

## Authors' contributions

SF designed the biomechanics study, developed equations of motion, collected and analyzed the data, and wrote and revised the draft. EA designed the psychoacoustic experiment, and wrote and revised the draft. HKa assisted acquisition, analysis, and interpretation of data of the psychoacoustic experiment. HKi wrote and revised the draft. All authors read, finalized, and approved the final manuscript.

## Supplementary Material

Additional file 1 **Equations of motion for inverse and forward dynamics computations**. The file contains complete equations of motion used for performing inverse and forward dynamics analyses. The upper extremity was assumed as four interconnected rigid links (upper-arm, forearm, hand, and finger). Click here for file
