# Supplementary material for: Control of multi-joint arm movements for the manipulation of touch in keystroke by expert pianists
Source: BMC Neurosci. 2010 Jul 14;11:82. doi: 10.1186/1471-2202-11-82 (PMC2919541; doi:10.1186/1471-2202-11-82)
Supplement: Additional file 1 — Equations of motion for inverse and forward dynamics computations. The file contains complete equations of motion used for performing inverse and forward dynamics analyses. The upper extremity was assumed as four interconnected rigid links (upper-arm, forearm, hand, and finger). [file 1471-2202-11-82-S1.PDF]

APPENDIX

Inverse dynamics computation

Basically, equations of motion of the four linked segment model can be written as follows.

$$\tau = \mathbf{I} \bullet \ddot{\mathbf{\Phi}} - \mathbf{VEL} - \mathbf{GRA} - \mathbf{REA}$$

$$\mathbf{I} = \begin{bmatrix} I_{11} & I_{12} & I_{13} & I_{14} \\ I_{21} & I_{22} & I_{23} & I_{24} \\ I_{31} & I_{32} & I_{33} & I_{34} \\ I_{41} & I_{42} & I_{43} & I_{44} \end{bmatrix}, \quad \mathbf{\Phi} = \begin{bmatrix} \phi_1 \\ \phi_2 \\ \phi_3 \\ \phi_4 \end{bmatrix}, \quad \mathbf{VEL} = \begin{bmatrix} V_1 \\ V_2 \\ V_3 \\ V_4 \end{bmatrix}, \quad \mathbf{GRA} = \begin{bmatrix} G_1 \\ G_2 \\ G_3 \\ G_4 \end{bmatrix}$$

$$\mathbf{REA} = \begin{bmatrix} J_{11} & J_{12} & J_{13} & J_{14} \\ J_{21} & J_{22} & J_{23} & J_{24} \end{bmatrix} \begin{bmatrix} F_x \\ F_y \end{bmatrix}$$

τ is the vector of muscle (residual) torque terms, $\mathbf{I}$ is the inertia matrix, $\mathbf{VEL}$ is the vector of centripetal and Coriolis terms, $\mathbf{GRA}$ is the vector of gravitational terms, $\mathbf{REA}$ is the vector of key-reaction force terms, and ϕ_i is joint angle (i = 1: shoulder, 2: elbow, 3: wrist, 4: finger (MP joint)). Each of these terms are described as follows

$$\begin{aligned} I_{11} = {} & I_1 + I_2 + I_3 + I_4 + m_1 r_1^2 + m_2 \left(l_1^2 + r_2^2\right) + m_3 \left(l_1^2 + l_2^2 + r_3^2\right) + m_4 \left(l_1^2 + l_2^2 + l_3^2 + r_4^2\right) \\ & + 2\left(m_2 r_2 l_1 + m_3 l_1 l_2 + m_4 l_1 l_2\right)\cos\phi_2 + 2\left(m_3 r_3 l_1 + m_4 l_1 l_3\right)\cos\left(\phi_2 + \phi_3\right) + 2\left(m_4 r_4 l_1\right)\cos\left(\phi_2 + \phi_3 + \phi_4\right) \\ & + 2\left(m_3 r_3 l_2 + m_4 l_2 l_3\right)\cos\phi_3 + 2\left(m_4 r_4 l_2\right)\cos\left(\phi_3 + \phi_4\right) + 2\left(m_4 r_4 l_3\right)\cos\phi_4 \end{aligned}$$

$$\begin{aligned} I_{12} = {} & I_2 + I_3 + I_4 + m_2 r_2^2 + m_3 \left(l_2^2 + r_3^2\right) + m_4 \left(l_2^2 + l_3^2 + r_4^2\right) \\ & + \left(m_2 r_2 l_1 + m_3 l_1 l_2 + m_4 l_1 l_2\right)\cos\phi_2 + \left(m_3 r_3 l_1 + m_4 l_1 l_3\right)\cos\left(\phi_2 + \phi_3\right) + \left(m_4 r_4 l_1\right)\cos\left(\phi_2 + \phi_3 + \phi_4\right) \\ & + 2\left(m_3 r_3 l_2 + m_4 l_2 l_3\right)\cos\phi_3 + 2\left(m_4 r_4 l_2\right)\cos\left(\phi_3 + \phi_4\right) + 2\left(m_4 r_4 l_3\right)\cos\phi_4 \end{aligned}$$

$$\begin{aligned} I_{13} = {} & I_3 + I_4 + m_3 r_3^2 + m_4 \left(l_3^2 + r_4^2\right) \\ & + \left(m_3 r_3 l_1 + m_4 l_1 l_3\right)\cos\left(\phi_2 + \phi_3\right) + \left(m_4 r_4 l_1\right)\cos\left(\phi_2 + \phi_3 + \phi_4\right) \\ & + \left(m_3 r_3 l_2 + m_4 l_2 l_3\right)\cos\phi_3 + \left(m_4 r_4 l_2\right)\cos\left(\phi_3 + \phi_4\right) + 2\left(m_4 r_4 l_3\right)\cos\phi_4 \end{aligned}$$

$$I_{14} = I_4 + m_4 r_4^2 + \left(m_4 r_4 l_3\right)\cos\phi_4 + \left(m_4 r_4 l_2\right)\cos\left(\phi_3 + \phi_4\right) + \left(m_4 r_4 l_1\right)\cos\left(\phi_2 + \phi_3 + \phi_4\right)$$

$$\begin{aligned} I_{21} = {} & I_2 + I_3 + I_4 + m_2 r_2^2 + m_3 \left(l_2^2 + r_3^2\right) + m_4 \left(l_2^2 + l_3^2 + r_4^2\right) \\ & + \left(m_2 r_2 l_1 + m_3 l_1 l_2 + m_4 l_1 l_2\right)\cos\phi_2 + \left(m_3 r_3 l_1 + m_4 l_1 l_3\right)\cos\left(\phi_2 + \phi_3\right) + \left(m_4 r_4 l_1\right)\cos\left(\phi_2 + \phi_3 + \phi_4\right) \\ & + 2\left(m_3 r_3 l_2 + m_4 l_2 l_3\right)\cos\phi_3 + 2\left(m_4 r_4 l_2\right)\cos\left(\phi_3 + \phi_4\right) + 2\left(m_4 r_4 l_3\right)\cos\phi_4 \end{aligned}$$

$$\begin{aligned} I_{22} = {} & I_2 + I_3 + I_4 + m_2 r_2^2 + m_3 \left(l_2^2 + r_3^2\right) + m_4 \left(l_2^2 + l_3^2 + r_4^2\right) + \\ & + 2\left(m_3 r_3 l_2 + m_4 l_2 l_3\right)\cos\phi_3 + 2\left(m_4 r_4 l_2\right)\cos\left(\phi_3 + \phi_4\right) + 2\left(m_4 r_4 l_3\right)\cos\phi_4 \end{aligned}$$

$$I_{23} = I_3 + I_4 + m_3 r_3^2 + m_4 \left(l_3^2 + r_4^2\right) + \left(m_3 r_3 l_2 + m_4 l_2 l_3\right)\cos\phi_3 + \left(m_4 r_4 l_2\right)\cos\left(\phi_3 + \phi_4\right) + 2\left(m_4 r_4 l_3\right)\cos\phi_4$$

$$I_{24} = I_4 + m_4 r_4^2 + \left(m_4 r_4 l_2\right)\cos\left(\phi_3 + \phi_4\right) + \left(m_4 r_4 l_3\right)\cos\phi_4$$

$$
\begin{aligned}
I_{31} = {} & I_3 + I_4 + m_3 r_3^{\,2} + m_4 \left(l_3^{\,2} + r_4^{\,2}\right) \\
& + \left(m_3 r_3 l_1 + m_4 l_1 l_3\right)\cos\left(\phi_2 + \phi_3\right) + \left(m_4 r_4 l_1\right)\cos\left(\phi_2 + \phi_3 + \phi_4\right) \\
& + \left(m_3 r_3 l_2 + m_4 l_2 l_3\right)\cos\phi_3 + \left(m_4 r_4 l_2\right)\cos\left(\phi_3 + \phi_4\right) + 2\left(m_4 r_4 l_3\right)\cos\phi_4
\end{aligned}
$$

$$
\begin{aligned}
I_{32} = {} & I_3 + I_4 + m_3 r_3^{\,2} + m_4 \left(l_3^{\,2} + r_4^{\,2}\right) \\
& + \left(m_3 r_3 l_2 + m_4 l_2 l_3\right)\cos\phi_3 + \left(m_4 r_4 l_2\right)\cos\left(\phi_3 + \phi_4\right) + 2\left(m_4 r_4 l_3\right)\cos\phi_4
\end{aligned}
$$

$$
I_{33} = I_3 + I_4 + m_3 r_3^{\,2} + m_4 \left(l_3^{\,2} + r_4^{\,2}\right) + 2\left(m_4 r_4 l_3\right)\cos\phi_4
$$

$$
I_{34} = I_4 + m_4 r_4^{\,2} + \left(m_4 r_4 l_3\right)\cos\phi_4
$$

$$
\begin{aligned}
I_{41} = {} & I_4 + m_4 r_4^{\,2} \\
& + \left(m_4 r_4 l_1\right)\cos\left(\phi_2 + \phi_3 + \phi_4\right) + \left(m_4 r_4 l_3\right)\cos\phi_4 + \left(m_4 r_4 l_2\right)\cos\left(\phi_3 + \phi_4\right)
\end{aligned}
$$

$$
\begin{aligned}
I_{42} = {} & I_4 + m_4 r_4^{\,2} \\
& + \left(m_4 r_4 l_2\right)\cos\left(\phi_3 + \phi_4\right) + \left(m_4 r_4 l_3\right)\cos\phi_4
\end{aligned}
$$

$$
I_{43} = I_4 + m_4 r_4^{\,2} + \left(m_4 r_4 l_3\right)\cos\phi_4
$$

$$
I_{44} = I_4 + m_4 r_4^{\,2}
$$

$$
\begin{aligned}
V_1 = {} & \dot\phi_2^{\,2}\left[\left(m_2 r_2 l_1 + m_3 l_1 l_2 + m_4 l_1 l_2\right)\sin\phi_2 + \left(m_3 r_3 l_1 + m_4 l_1 l_3\right)\sin\left(\phi_2 + \phi_3\right) + \left(m_4 r_4 l_1\right)\sin\left(\phi_2 + \phi_3 + \phi_4\right)\right] \\
& + \dot\phi_3^{\,2}\begin{bmatrix} \left(m_3 r_3 l_1 + m_4 l_1 l_3\right)\sin\left(\phi_2 + \phi_3\right) + \left(m_4 r_4 l_1\right)\sin\left(\phi_2 + \phi_3 + \phi_4\right) \\ + \left(m_3 r_3 l_2 + m_4 l_2 l_3\right)\sin\phi_3 + \left(m_4 r_4 l_2\right)\sin\left(\phi_3 + \phi_4\right) \end{bmatrix} \\
& + \dot\phi_4^{\,2}\left[\left(m_4 r_4 l_3\right)\sin\phi_4 + \left(m_4 r_4 l_2\right)\sin\left(\phi_3 + \phi_4\right) + \left(m_4 r_4 l_1\right)\sin\left(\phi_2 + \phi_3 + \phi_4\right)\right] \\
& + \dot\phi_1\,\dot\phi_2\left[2\left(m_2 r_2 l_1 + m_3 l_1 l_2 + m_4 l_1 l_2\right)\sin\phi_2 + 2\left(m_3 r_3 l_1 + m_4 l_1 l_3\right)\sin\left(\phi_2 + \phi_3\right) + 2\left(m_4 r_4 l_1\right)\sin\left(\phi_2 + \phi_3 + \phi_4\right)\right] \\
& + \dot\phi_1\,\dot\phi_3\begin{bmatrix} 2\left(m_3 r_3 l_2 + m_4 l_2 l_3\right)\sin\phi_3 + 2\left(m_3 r_3 l_1 + m_4 l_1 l_3\right)\sin\left(\phi_2 + \phi_3\right) + 2\left(m_4 r_4 l_2\right)\sin\left(\phi_3 + \phi_4\right) \\ + 2\left(m_4 r_4 l_1\right)\sin\left(\phi_2 + \phi_3 + \phi_4\right) \end{bmatrix} \\
& + \dot\phi_1\,\dot\phi_4\left[2\left(m_4 r_4 l_3\right)\sin\phi_4 + 2\left(m_4 r_4 l_2\right)\sin\left(\phi_3 + \phi_4\right) + 2\left(m_4 r_4 l_1\right)\sin\left(\phi_2 + \phi_3 + \phi_4\right)\right] \\
& + \dot\phi_2\,\dot\phi_3\begin{bmatrix} 2\left(m_3 r_3 l_2 + m_4 l_2 l_3\right)\sin\phi_3 + 2\left(m_3 r_3 l_1 + m_4 l_1 l_3\right)\sin\left(\phi_2 + \phi_3\right) + 2\left(m_4 r_4 l_2\right)\sin\left(\phi_3 + \phi_4\right) \\ + 2\left(m_4 r_4 l_1\right)\sin\left(\phi_2 + \phi_3 + \phi_4\right) \end{bmatrix} \\
& + \dot\phi_2\,\dot\phi_4\left[2\left(m_4 r_4 l_3\right)\sin\phi_4 + 2\left(m_4 r_4 l_2\right)\sin\left(\phi_3 + \phi_4\right) + 2\left(m_4 r_4 l_1\right)\sin\left(\phi_2 + \phi_3 + \phi_4\right)\right] \\
& + \dot\phi_3\,\dot\phi_4\left[2\left(m_4 r_4 l_3\right)\sin\phi_4 + 2\left(m_4 r_4 l_2\right)\sin\left(\phi_3 + \phi_4\right) + 2\left(m_4 r_4 l_1\right)\sin\left(\phi_2 + \phi_3 + \phi_4\right)\right]
\end{aligned}
$$

$$
\begin{aligned}
V_2 = {} & -\dot\phi_1^{\,2}\left[\left(m_2 r_2 l_1 + m_3 l_1 l_2 + m_4 l_1 l_2\right)\sin\phi_2 + \left(m_3 r_3 l_1 + m_4 l_1 l_3\right)\sin\left(\phi_2 + \phi_3\right) + \left(m_4 r_4 l_1\right)\sin\left(\phi_2 + \phi_3 + \phi_4\right)\right] \\
& + \dot\phi_3^{\,2}\left[\left(m_3 r_3 l_2 + m_4 l_2 l_3\right)\sin\phi_3 + \left(m_4 r_4 l_2\right)\sin\left(\phi_3 + \phi_4\right)\right] \\
& + \dot\phi_4^{\,2}\left[\left(m_4 r_4 l_3\right)\sin\phi_4 + \left(m_4 r_4 l_2\right)\sin\left(\phi_3 + \phi_4\right)\right] \\
& + \dot\phi_1\,\dot\phi_3\left[2\left(m_3 r_3 l_2 + m_4 l_2 l_3\right)\sin\phi_3 + 2\left(m_4 r_4 l_2\right)\sin\left(\phi_3 + \phi_4\right)\right] \\
& + \dot\phi_1\,\dot\phi_4\left[2\left(m_4 r_4 l_3\right)\sin\phi_4 + 2\left(m_4 r_4 l_2\right)\sin\left(\phi_3 + \phi_4\right)\right] \\
& + \dot\phi_2\,\dot\phi_3\left[2\left(m_3 r_3 l_2 + m_4 l_2 l_3\right)\sin\phi_3 + 2\left(m_4 r_4 l_2\right)\sin\left(\phi_3 + \phi_4\right)\right] \\
& + \dot\phi_2\,\dot\phi_4\left[2\left(m_4 r_4 l_3\right)\sin\phi_4 + 2\left(m_4 r_4 l_2\right)\sin\left(\phi_3 + \phi_4\right)\right] \\
& + \dot\phi_3\,\dot\phi_4\left[2\left(m_4 r_4 l_3\right)\sin\phi_4 + 2\left(m_4 r_4 l_2\right)\sin\left(\phi_3 + \phi_4\right)\right]
\end{aligned}
$$

$$V_3 = -\dot{\phi}_1^{\,2}\begin{bmatrix}(m_3r_3l_1+m_4l_1l_3)\sin(\phi_2+\phi_3)+(m_4r_4l_1)\sin(\phi_2+\phi_3+\phi_4)\\ +(m_3r_3l_2+m_4l_2l_3)\sin\phi_3+(m_4r_4l_2)\sin(\phi_3+\phi_4)\end{bmatrix}$$
$$-\dot{\phi}_2^{\,2}\left[(m_3r_3l_2+m_4l_2l_3)\sin\phi_3+(m_4r_4l_2)\sin(\phi_3+\phi_4)\right]$$
$$+\dot{\phi}_4^{\,2}\left[(m_4r_4l_3)\sin\phi_4\right]$$
$$-\dot{\phi}_1\,\dot{\phi}_2\left[2(m_3r_3l_2+m_4l_2l_3)\sin\phi_3+2(m_4r_4l_2)\sin(\phi_3+\phi_4)\right]$$
$$+\dot{\phi}_1\,\dot{\phi}_4\left[2(m_4r_4l_3)\sin\phi_4\right]$$
$$+\dot{\phi}_2\,\dot{\phi}_4\left[2(m_4r_4l_3)\sin\phi_4\right]$$
$$+\dot{\phi}_3\,\dot{\phi}_4\left[2(m_4r_4l_3)\sin\phi_4\right]$$

$$V_4 = -\dot{\phi}_1^{\,2}\begin{bmatrix}(m_4r_4l_1)\sin(\phi_2+\phi_3+\phi_4)\\ +(m_4r_4l_3)\sin\phi_4+(m_4r_4l_2)\sin(\phi_3+\phi_4)\end{bmatrix}$$
$$-\dot{\phi}_2^{\,2}\left[(m_4r_4l_3)\sin\phi_4+(m_4r_4l_2)\sin(\phi_3+\phi_4)\right]$$
$$-\dot{\phi}_3^{\,2}\left[(m_4r_4l_3)\sin\phi_4\right]$$
$$-\dot{\phi}_1\,\dot{\phi}_2\left[2(m_4r_4l_3)\sin\phi_4+2(m_4r_4l_2)\sin(\phi_3+\phi_4)\right]$$
$$-\dot{\phi}_1\,\dot{\phi}_3\left[2(m_4r_4l_3)\sin\phi_4\right]$$
$$-\dot{\phi}_2\,\dot{\phi}_3\left[2(m_4r_4l_3)\sin\phi_4\right]$$

$$G_1 = -g\begin{bmatrix}(m_1r_1+m_2l_1+m_3l_1+m_4l_1)\cos\phi_1+(m_2r_2+m_3l_2+m_4l_2)\cos(\phi_1+\phi_2)+(m_3r_3+m_4l_3)\cos(\phi_1+\phi_2+\phi_3)\\ +(m_4r_4)\cos(\phi_1+\phi_2+\phi_3+\phi_4)\end{bmatrix}$$
$$G_2 = -g\left[(m_2r_2+m_3l_2+m_4l_2)\cos(\phi_1+\phi_2)+(m_3r_3+m_4l_3)\cos(\phi_1+\phi_2+\phi_3)+(m_4r_4)\cos(\phi_1+\phi_2+\phi_3+\phi_4)\right]$$
$$G_3 = -g\left[(m_3r_3+m_4l_3)\cos(\phi_1+\phi_2+\phi_3)+(m_4r_4)\cos(\phi_1+\phi_2+\phi_3+\phi_4)\right]$$
$$G_4 = -g\left[(m_4r_4)\cos(\phi_1+\phi_2+\phi_3+\phi_4)\right]$$
$$J_{11} = l_1\sin\phi_1+l_2\sin(\phi_1+\phi_2)+l_3\sin(\phi_1+\phi_2+\phi_3)+l_4\sin(\phi_1+\phi_2+\phi_3+\phi_4)$$
$$J_{21} = l_2\sin(\phi_1+\phi_2)+l_3\sin(\phi_1+\phi_2+\phi_3)+l_4\sin(\phi_1+\phi_2+\phi_3+\phi_4)$$
$$J_{31} = l_3\sin(\phi_1+\phi_2+\phi_3)+l_4\sin(\phi_1+\phi_2+\phi_3+\phi_4)$$
$$J_{41} = l_4\sin(\phi_1+\phi_2+\phi_3+\phi_4)$$
$$J_{12} = l_1\cos\phi_1+l_2\cos(\phi_1+\phi_2)+l_3\cos(\phi_1+\phi_2+\phi_3)+l_4\cos(\phi_1+\phi_2+\phi_3+\phi_4)$$
$$J_{22} = l_2\cos(\phi_1+\phi_2)+l_3\cos(\phi_1+\phi_2+\phi_3)+l_4\cos(\phi_1+\phi_2+\phi_3+\phi_4)$$
$$J_{32} = l_3\cos(\phi_1+\phi_2+\phi_3)+l_4\cos(\phi_1+\phi_2+\phi_3+\phi_4)$$
$$J_{42} = l_4\cos(\phi_1+\phi_2+\phi_3+\phi_4)$$

SYMBOLS. I_i = moment of inertia about the center of gravity, r_i = distance to center of gravity from proximal joint of the segment, l_i = length, m_i = mass (i = 1: upper arm, 2: forearm, 3: hand, 4: finger). The hand was defined as a portion from the wrist joint center to MP joint center, while the finger was from the MP joint center to the fingertip. The tangential force was set to nil for simplicity of computation.

We then computed the **MUS** that removed static component counteracting with gravitational torque (**GRA**)

from the muscular torque (τ) in order to determine the dynamic component directly used for limb movement production (i.e. $\mathbf{MUS} = \tau + \mathbf{GRA}$).

Forward dynamics computation

Using the value of computed muscular, velocity-dependent, and reaction-force-dependent torques, joint angular acceleration attributed to these torques can be computed as follows.

$$\ddot{\mathbf{\Theta}} = \mathbf{I}^{-1}(\mathbf{MUS} + \mathbf{VEL} + \mathbf{REA})$$

where, $\ddot{\mathbf{\Theta}}$ represents angular acceleration produced by individual torque at the shoulder, elbow, wrist, and finger joint.
